# An Effective Test (EOmciSS) for Screening Older Adults With Mild Cognitive Impairment in a Community Setting: Development and Validation Study

**DOI:** 10.2196/40858

**Published:** 2023-01-30

**Authors:** Jingsong Wu, Jingnan Tu, Zhizhen Liu, Lei Cao, Youze He, Jia Huang, Jing Tao, Mabel N K Wong, Lidian Chen, Tatia M C Lee, Chetwyn C H Chan

**Affiliations:** 1 College of Rehabilitation Medicine Fujian University of Traditional Chinese Medicine Fuzhou China; 2 The Academy of Rehabilitation Industry Fujian University of Traditional Chinese Medicine Fuzhou China; 3 National-Local Joint Engineering Research Center of Rehabilitation Medicine Technology Fujian University of Traditional Chinese Medicine Fuzhou China; 4 Fujian Key Laboratory of Rehabilitation Technology Fujian University of Traditional Chinese Medicine Fuzhou China; 5 Department of Psychology The Education University of Hong Kong Hong Kong Hong Kong; 6 Department of Rehabilitation Sciences The Hong Kong Polytechnic University Hong Kong Hong Kong; 7 Laboratory of Neuropsychology The University of Hong Kong Hong Kong Hong Kong

**Keywords:** mild cognitive impairment, digital assessment, digital health, community dwelling, screening test, older adults, aging

## Abstract

**Background:**

Early detection of mild cognitive impairment (MCI) symptoms is an important step to its diagnosis and intervention. We developed a new screening test called “Efficient Online MCI Screening System” (EOmciSS) for use in community-dwelling older adults. It is a self-paced cognitive test to be completed within 10 minutes on tablets or smartphones in homes or care centers for older adults.

**Objective:**

This study aims to test the validity of EOmciSS for identifying community-dwelling older adults with MCI risks.

**Methods:**

Participants (N=827) completed EOmciSS and other screening tests for MCI. The psychometric properties tested were “subscale item difficulty,” “discriminative index,” “internal consistency,” and “construct validity.” We also tested between-group discrimination using the cross-validation method in an MCI group and a normal cognitive function (NCF) group.

**Results:**

A total of 3 accuracy factors and 1 reaction time factor explained the structure of the 20 item factors. The difficulty level of accuracy factors (ie, “trail making,” “clock drawing,” “cube copying,” “delayed recall”) was 0.63-0.99, whereas that of the reaction time factor was 0.77-0.95. The discriminative index of the medium-to-high-difficulty item factors was 0.39-0.97. The internal consistency (Cronbach α) ranged from .41 (for few item factors) to .96. The training data set contained 9 item factors (CC-Acc1, *P*<.001; CD-Acc1, *P*=.07; CD-Acc2, *P*=.06; CD-Acc3, *P*<.001; TM-Acc4, *P*=.07; DR-Acc1, *P*=.03; RS, *P*=.06; DR-RT1, *P*=.02; and DR-RT2, *P*=.05) that were significant predictors for an MCI classification versus NCF classification. Depressive symptoms were identified as significant factors (*P*<.001) influencing the performance of participants, and were an integral part of our test system. Age (*P*=.15), number of years of education (*P*=.18), and proficiency in using an electronic device (*P*=.39) did not significantly influence the scores nor classification of participants. Application of the MCI/NCF cutoff score (7.90 out of 9.67) to the validation data set yielded an area under the curve of 0.912 (*P*<.001; 95% CI 0.868-0.955). The sensitivity was 84.9%, specificity was 85.1%, and the Youden index was 0.70.

**Conclusions:**

EOmciSS was valid and reliable for identifying older adults with significant risks of MCI. Our results indicate that EOmciSS has higher sensitivity and specificity than those of the Computer-Administered Neuropsychological Screen for Mild Cognitive Impairment and the Computerized Cognitive Screen. The user interface, online operation, and self-paced format allowed the test system to be operated by older adults or their caregivers in different settings (eg, home or care centers for older adults). Depressive symptoms should be an integral part in future MCI screening systems because they influence the test performance and, hence, MCI risk.

**Trial Registration:**

Chinese Clinical Trial Registry ChiCTR2000039411; http://www.chictr.org.cn/showprojen.aspx?proj=62903

## Introduction

Approximately 50 million people worldwide suffer from dementia, and this number is expected to increase to 152 million by 2050 [1]. Among older adults (≥60 years), the prevalence of dementia is about 5%-7% [2], which brings heavy burdens to families, society, and health care systems [3]. Studies have shown that early detection of cognitive impairment and intervention can delay the progression from mild cognitive impairment (MCI) to dementia [4].

Early detection of MCI symptoms in the community-dwelling older population is the first step of this endeavor [5]. Studies have reported comparable validity between paper-and-pencil methods and computerized methods in MCI screening [6].

Screening tests can be in various forms: self-evaluation, evaluation by an examiner, or evaluation by a caregiver. Self-rating tests rely on self-reporting by the individual [7], and an example is the Ascertain Dementia-8 (AD8) questionnaire. The AD8 questionnaire has been reported to have an internal consistency of .66 (Cronbach α), with a sensitivity of 80% and specificity of 59% [8]. The questionnaire takes about 3 minutes to complete and is used mainly to screen dementia rather than MCI risk [9,10].

Performance tests involve the administration and scoring of the test by a medical professional. An example is the Montreal Cognitive Assessment (MoCA). The internal consistency of the MoCA is .83 (Cronbach α) [11]. A systematic review reported that the MoCA had a sensitivity of 83.9% and specificity of 74.6% for distinguishing individuals with MCI from those who were considered to have normal cognitive function (NCF). The time needed to administer the MoCA is 10–15 minutes [12]. Scholars have commented that the MoCA score is affected by the education level of the individual being tested [13] and that the score should be rated by a medical professional [14].

The General Practitioner Assessment of Cognition (GPCOG) uses the caregiver as the informant in addition to professional scoring. The internal consistency of the GPCOG has been reported to be .84 (Cronbach α), with a sensitivity of 82% and specificity of 83% [15]. The GPCOG takes less than 10 minutes to administer [16-18].

Apart from paper-and-pencil tests, computerized screening tests or digital screening tests take advantage of information technology and the portability of service delivery [19,20]. For instance, the Computer-Administered Neuropsychological Screen for Mild Cognitive Impairment (CANS-MCI) [21] covers comprehensive cognitive functions. It had a sensitivity of 81% and specificity of 73% for differentiating MCI from individuals with NCF. The CANS-MCI takes about 30 minutes to complete. By contrast, the Computerized Cognitive Screen (CoCoSc) [22] requires 15 minutes for completion, but has a lower ability (78% sensitivity and 69% specificity) to differentiate individuals with impaired cognition from those with normal cognition. One crucial factor that may confound individuals’ performance is their emotional state, particularly a depressive mood. The latter has been found to be a common comorbidity of MCI [23]. Depressive symptoms (eg, lack of motivation, slowness to respond) would contribute to the false-positive rate of test results [24-26]. However, the test of a depressive mood is not a feature in the aforementioned computerized screening tests.

Given the aforesaid unmet needs and shortcomings, we developed a new screening test called “Efficient Online MCI Screening System” (EOmciSS) for use by community-dwelling older adults. First, we selected test items of relatively high sensitivity and high specificity for MCI. “cube copying” (CC) and “clock drawing” (CD) have shown a sensitivity of 65.6% and 57.1% and specificity of 53.3% and 70.0%, respectively, for individuals with MCI versus individuals with NCF [27-29]. The “trail making” (TM) item has yielded a sensitivity of 51.8% and specificity of 80.2% for differentiating individuals with MCI from those with NCF [28]. The “reaction speed” (RS) item [30] has shown an accuracy (Acc) of 80.6% for individuals with MCI versus individuals with dementia. The “delayed recall” (DR) item has been shown to have a sensitivity of 83% and specificity of 65% for MCI versus Alzheimer disease in older adults [31]. For a depressive mood, the 15-item Geriatric Depression Scale (GDS-15; cutoff score ≥8 points) has been shown to yield an internal consistency of .793 (Cronbach α) in an older population [32].

EOmciSS has certain features that cater to the needs and self-paced operations of older users. For example, test instructions are delivered audially and visually simultaneously. Cross-audiovisual presentations can enhance the attention and, hence, the learning of users to understand the operation of test tasks [33]. The locations and times of all responses (eg, TM, CD, CC items) are recorded by pressing a touchscreen and can improve the prediction model.

We aimed to gather evidence of the validity of EOmciSS for identifying community-dwelling older adults with MCI risks. It is a self-paced, rapid, cognitive screening test for users that can be completed within 10 minutes on tablets or smartphones in homes or care centers. We hypothesized that the test items of EOmciSS would contribute significantly to identify individuals with a high risk of MCI, and that the sensitivity and specificity of EOmciSS would be comparable with those of other paper-and-pencil tests for MCI screening.

## Methods

### Inclusion Criteria

The inclusion criteria were as follows: (1) age between 55 and 75 years; (2) community dwelling and attending an activity center regularly; (3) had received education for more than 6 years [34]; and (4) having normal vision and hearing with/without corrective devices.

### Exclusion Criteria

The exclusion criteria were taking medications (eg, antidepressant, sedative) in the previous 2 weeks or a known history of (1) brain trauma, brain tumor, cerebral infarction, cerebral hemorrhage, intracranial infection, Parkinson, epilepsy, or other neurological diseases; (2) depression, anxiety, schizophrenia, or other mental illnesses; (3) abuse of alcohol or other substances.

### Participants

Based on convenience sampling, participants (older adults) were recruited from 32 community centers for older adults in a city in China from June 2019 to December 2020.

A total of 1081 participants were recruited and completed testing. Among them, 196 were verified to have received ≤6 years of education. Another 58 participants were found to have excessive amounts of missing data. The final sample size entering the analyses was 827 (Multimedia Appendix 1).

### Classification of the MCI Versus NC Groups

Classification of participants in the MCI group was based on the diagnostic criteria published by Petersen [35] and the MCI guidelines set by the American Academy of Neurology in 2018 [36]. There were 4 criteria. The first criterion was subjective reporting of memory deficits by the participants or their informant. The second criterion was a score of 19-24 on the Chinese (Fuzhou) version of the MoCA (C-MoCA). This is an objective measure [34,37] that indicates MCI. The cutoff score of 24 (as compared with 26 in the original MoCA [11]) has been validated for use among the Chinese population. The third criterion was a score greater than 2 minus standard deviations (ie, > –2SDs) on the Lawton Instrumental Activities of Daily Living (IADL) scale [38], which indicates the intact ability of the activities of daily living. The fourth criterion was 1 point or less on the AD8 questionnaire [8], which excludes the possibility of dementia. The criteria for classifying participants in the NCF group were a score of 25 or over on the C-MoCA and 1 point or less on the AD8 questionnaire, and greater than −2SDs on the IADL scale. According to these criteria, 579 participants belonged to the NCF group and 248 to the MCI group.

We adopted the diagnostic criteria stipulated by the National Institute on Aging-Alzheimer’s Association [39] to exclude participants who showed dementia symptoms. The criteria were (1) score of 18 or less on the C-MoCA [37]; (2) less than −2SDs on the IADL scale [38]; and (3) 2 points or more on the AD8 questionnaire [8].

### Procedures

Research assistants helped participants to access EOmciSS using a tablet. Participants began to complete the test with assistance from the instructions and sample items provided by EOmciSS. Research assistants did not aid the participants at any point during the test. Each participant yielded a set of EOmciSS item scores and a machine-generated total score. After completing EOmciSS, participants were administered a series of criterion tests: MoCA, AD8, and IADL. These tests were administered by a different group of research assistants who received training on delivering the 3 tests using standardized procedures. Participants were also assessed by physicians within our research team who had the experience of diagnosing MCI in their clinical practices.

### EOmciSS

There are 4 main features in the conceptualization of EOmciSS that facilitate large-scale screening in a community setting. First, the composition of the test contents takes an eclectic approach. The constructs of all the test items have been supported with sufficient evidence for differentiating individuals with MCI from individuals with NCF. These are “depressive mood,” “visual attention and flexibility,” “visuospatial and executive functions,” “memory and cognitive processing speed.” Second, the user interface is simple and allows for self-administration and the test to be completed at different times in different places. Third, the time required for most users to complete the test should be 10 minutes or less. Fourth, administration of the test and scoring are automated.

**Figure 1:**
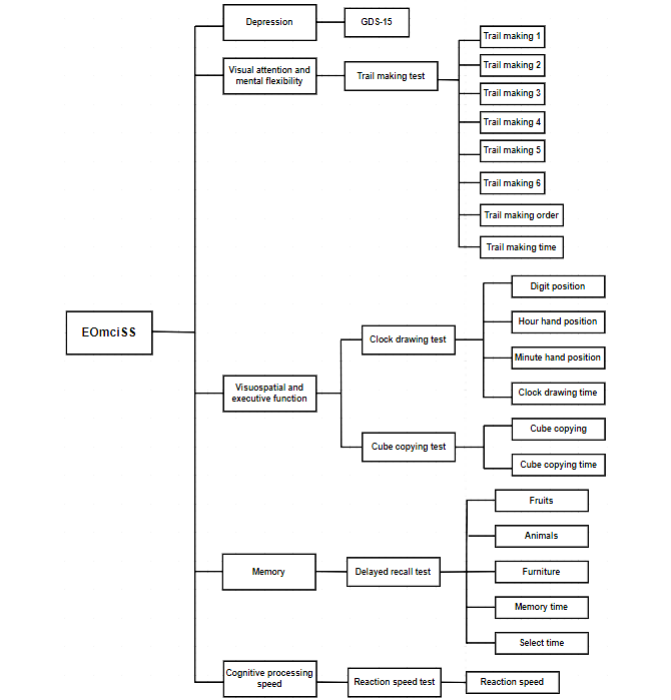
Test contents of EOmciSS. The test contents of EOmciSS cover 5 subtypes with 20 test items. The subtypes are “depression,” “visual attention and mental flexibility,” “visuospatial and executive functions,” “memory,” and “cognitive processing speed.” EOmciSS: Efficient Online MCI Screening System; GDS-15: 15-item Geriatric Depression Scale.

**Figure 2:**
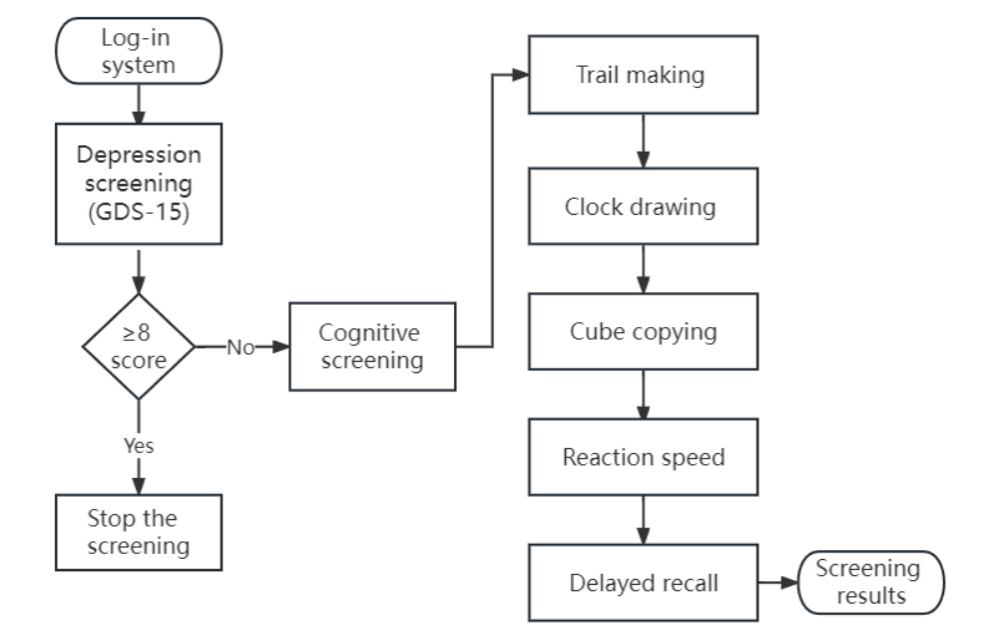
EOmciSS workflow. The EOmciSS workflow starts from the “log-in system” to completion of the screening test with printing of the result. The entire workflow is to be completed within 10 minutes. EOmciSS: Efficient Online MCI Screening System; GDS-15: 15-item Geriatric Depression Scale.

EOmciSS has 2 sections delivered in sequence (Figure 1). The first section is the first-level screen, which requires participants to complete the GDS-15 [40] for depressive symptoms. The Chinese version of the GDS-15 has 15 items, and a positive response to 1 item scores 1 point. The total score is 15 points, with 8 points or more [41,42] being the cutoff for indicating a possible depressive mood. Individuals who score 8 points or more are advised to consult medical practitioners, and the assessment is terminated. The aim of the first-level screen is to prevent depressive symptoms (eg, slow processing time) to confound the results of the subsequent items in the cognitive test. Participants who pass the screening for depressive symptoms proceed to the second-level screen, which is the cognitive test (Figure 2).

**Figure 3:**
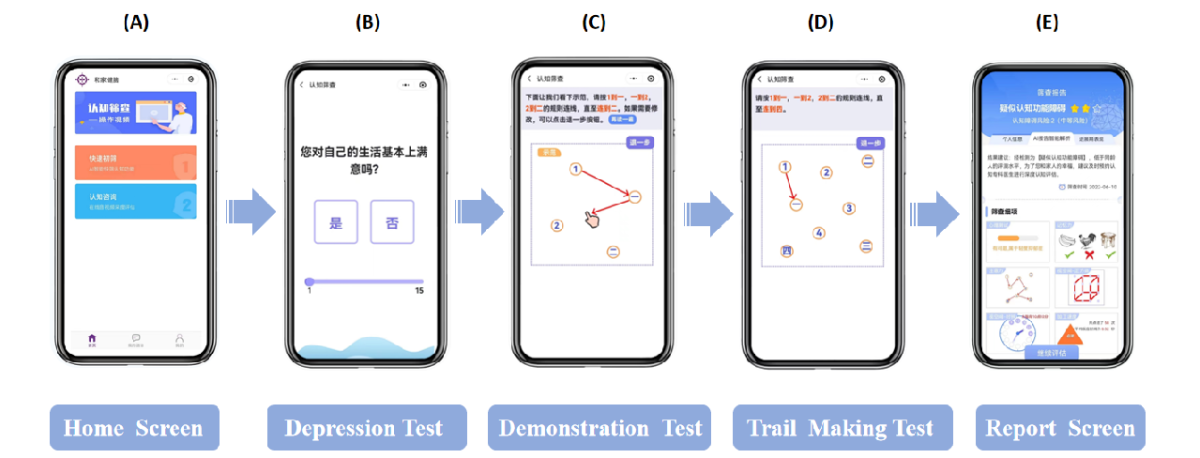
Screenshot examples of EOmciSS. Screenshot examples of EOmciSS showing: (A) the login page, (B) a “depressive mood” item (GDS-15), (C) the demonstration page of a test item, (D) beginning of the “Trail Making” subscale, and (E) the printing page of test results. EOmciSS: Efficient Online MCI Screening System; GDS-15: 15-item Geriatric Depression Scale.

The cognitive test has 5 subtests that contain 20 test items (Figure 1). The 20 test items comprise 14 Acc and 6 reaction time (RT) measures. The 5 subtests are the TM (8 items; 6 Acc + 1 RT measure, and 1 order), CD (4 items; 3 Acc + 1 RT measure), CC (2 items; 1 Acc + 1 RT measure), DR (5 items; 3 Acc + 2 RT measures), and RS (1 item; 1 RT measure). In each subtest, participants view the information and instructions of the test procedures in synchronized visual and audio formats (Figure 3). There are also sample items to enable participants to familiarize themselves with the test procedure and response sets. Participants make responses by hitting the icons that appear on the touchscreen of a tablet, a smartphone, or another type of electronic device. Depending on the test items, responses are quantified according to Acc and RT (Multimedia Appendix 2).

### External Criterion Measures

A total of 3 measures were used as the external criteria for establishing evidence of the concurrent validity of EOmciSS. The C-MoCA [43] contains 11 subtests; 8 of them cover different cognitive domains, including “visuospatial and executive functions,” “naming,” “instantaneous memory (not scored),” “attention,” “language,” “abstraction,” “delayed recall,” and “orientation.” The AD8 questionnaire is a short test for dementia that is sensitive to early changes in cognitive function [44]. The cutoff scores of the MoCA and AD8 for MCI that we adopted are stated above. The IADL scale measures the independence of daily living [45]. It contains 8 items on the performance levels (0-4 points) in different aspects of managing daily living tasks. A higher score reflects a higher level of independence.

### Data Analyses

Different methods were used to generate evidence on the validity of the 20 test items under EOmciSS subscales. At the test level, the structure of the 20 item factors (13 Acc + 6 RT + 1 order) was explored by confirmatory factor analysis. At item and subtest levels, “item difficulty,” “discriminative index,” and “internal consistency” were the indices. “Item difficulty” refers to the proportion of correct answers per item [46]. “Discriminative index” is the correlation between 1 item and its subtest score [47]. “Internal consistency” is Cronbach α computed for each of the 5 subtests.

As suggested by Boateng et al [46], known-group differentiation was used to estimate the sensitivity and specificity of test items to differentiate participants with MCI from those with NCF. The known-group labels of MCI and NCF were based on participants’ scores on the MoCA, AD8, and IADL. The validity of between-group discrimination was tested further with cross validation [48] by dividing participants into 2 subsets: the MCI group and the NCF group. The 70% (579/827, 70.1%) subset was the training set. The 30% (248/827, 29.9%) subset was the validation set. Between-subset differences in demographic characteristics were tested with the Student *t* test, Spearman rank correlation test, or *χ*^2^ test. Multivariate binary logistic regression was applied to test the significance of the 20 item factors for predicting the membership of participants in the NCF group or the MCI group. The area under the curve (AUC) and Hosmer-Lemeshow goodness-of-fit test were used to describe the ability of the regression model to differentiate MCI from NCF. The regression model obtained from the training set was tested with the sample in the validation set. The β coefficients yielded became the item weights of each significant item factor for computing the total score. Predicted memberships were compared with observed memberships to derive the optimal cutoff score for MCI and NCF, as well as its sensitivity, specificity, Youden index, and receiver operating characteristic curve.

### Ethical Approval of the Study Protocol

The Medical Ethics Committee of the Affiliated Rehabilitation Hospital of Fujian University of Traditional Chinese Medicine (Fuzhou, China) approved the study protocol (approval number 2019KY-002-02). The purposes of the study were explained to all participants, who provided written informed consent.

## Results

### Construct Validity

**Figure 4:**
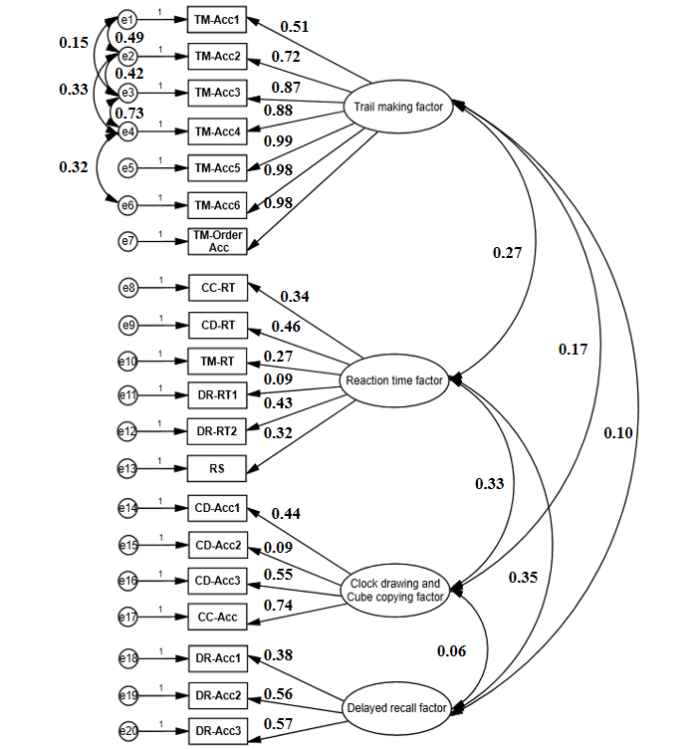
Path diagram showing the estimated parameter loadings from confirmatory factor analysis. A 4-factor structure is revealed on the accuracy, reaction time, and order factors for the 20 test items of EOmciSS. Acc: accuracy; CC: cube copying; CD: clock drawing; DR: delayed recall; EOmciSS: Efficient Online MCI Screening System; RT: reaction time; RS: reaction speed; TM: trail making.

A total of 20 item factors made up the performance Acc (n=14) and RT (n=6): TM-Acc (#1-6, #7 order), CD-Acc (#8-10), CC-Acc (#11), DR-Acc (#12-14), CC-RT (#15), CD-RT (#16), TM-RT (#17), DR-RT1 (#18), DR-RT2 (#19), and RS (#20). Confirmatory factor analysis revealed the Kaiser-Meyer-Olkin value to be 0.841, and the Bartlett test showed a significant difference (*P*<.001). Comparative fit index, standardized root-mean-squared residual, and root-mean-square error of approximation were 0.97, 0.007, and 0.048, respectively [49]. These results suggested a 4-factor structure for grouping the 20 item factors (Figure 4).

The first factor appeared to cover the Acc of the responses on TM items, and was called the “TM factor.” The second factor appeared to account for the Acc of the responses on the CD and CC items, and was called the “CD+CC factor.” The third factor appeared to cover the Acc of the responses on the DR items, and was called the “DR factor.” The fourth factor covered the CC-RT, CD-RT, TM-RT, DR-RT1, DR-RT2, and RS items, and was called the “RT factor.” This factor structure appeared to be consistent with the constructs of visual attention and mental flexibility (TM), visuospatial and executive function (CD and CC), memory (DR), and cognitive processing speed (RS).

The difficulty level of the items under the TM, CD+CC, DR, and RT subtests was 0.69-0.90, 0.63-0.99, 0.95-0.98, and 0.77-0.95, respectively. The corresponding discriminative index was 0.626-0.974, 0.224-0.886, 0.530-0.790, and 0.25-0.60 (Table 1). The internal consistency index (Cronbach α) was .959, .483, .473, and .411 for the TM factor, CD+CC factor, DR factor, and RT factor, respectively.

**Table 1:** Item difficulties (mean values) and discriminative indices (item total correlations) of the 20 item factors and internal consistencies (Cronbach α) of the 4 subscales of EOmciSS^a^.^b^

EOmciSS item factors		Item difficulty	Discriminative index	Internal consistency (Cronbach α)
**Trail making factor**				.959
	TM^c^-Acc1	0.90	0.626	
	TM-Acc2	0.83	0.797	
	TM-Acc3	0.76	0.905	
	TM-Acc4	0.75	0.917	
	TM-Acc5	0.70	0.968	
	TM-Acc6	0.71	0.957	
	TM-order-Acc	0.69	0.974	
**Clock drawing and cube copying factor**				.483
	CD^d^-Acc1	0.99	0.224	
	CD-Acc2	0.96	0.392	
	CD-Acc3	0.85	0.649	
	CC-Acc1	0.63	0.886	
**Delayed recall factor**				.473
	DR^e^-Acc1	0.95	0.790	
	DR-Acc2	0.98	0.530	
	DR-Acc3	0.97	0.611	
	CC^f^-RT	0.88	0.49	
	CD-RT	0.88	0.49	
	TM-RT	0.89	0.45	
	DR-RT1	0.95	0.25	
	DR-RT2	0.89	0.47	
**Reaction time factor**				.411
	RS^g^	0.77	0.60	

^a^EOmciSS: Efficient Online MCI Screening System.

^b^The 4 subscales were grouped based on the results of the confirmatory factor analysis.

^c^TM: trail making.

^d^CD: clock drawing.

^e^DR: delayed recall.

^f^CC: cube copying.

^g^RS: reaction speed.

### Discriminative Validity (Training Set)

There were 402 participants with NCF and 177 participants with MCI in the training set (Table 2). There were 175 participants with NCF and 73 participants with MCI in the validation set. The percentage of participants with MCI in the total sample for the training and validation sets was 30.6% (177/579) and 29.4% (73/248), respectively, and this difference was not significant (*P*=.74). In the training set, the MCI subgroup was significantly older (*P*=.048), had fewer years of education (*P*<.001), and spent less time using an electronic device (*P*<.001) than the NCF subgroup (Table 2). Other between-subgroup demographic variables did not show significant differences (*P*=.68, .89, .12, .68, and .39 for sex, BMI, concomitant chronic disease, smoking, and drinking, respectively). Participants in the NCF subgroup showed a significantly higher mean score on the MoCA (*P*<.001) and a lower mean score on the GDS-15 (*P*<.001) than those in the MCI subgroup. Significant differences were not found in the mean score for the IADL scale (*P*=.24).

Participants with NCF in the training set showed significantly higher Acc than those with MCI in all EOmciSS item factors (*χ*^2^_1_=7.52-127.75, *P*<.001). Participants with NCF showed faster RT on correct items than those with MCI across all item factors (Z=5.28-7.33, *P*<.001).

The 20 item factors were the predictor variables. Age, education, score on the GDS-15, and proficiency using an electronic device were covariates. The regression model contained 4 item factors (CC-Acc1, *P*<.001; CD-Acc3, *P*<.001; DR-Acc1, *P*=.03; and DR-RT1, *P*=.02) as the significant predictor at *P*<.05; 5 item factors were marginally significant predictors at *P*=.05-.07 (Table 3). The score on the GDS-15 was the only significant (*P*<.001) covariate in the model. The AUC of the MCI prediction model versus the NCF prediction model was 0.908 (95% CI 0.881-0.934; *P*<.001), which was over 0.75 and suggested satisfactory discriminative power. The Hosmer-Lemeshow goodness-of-fit test also indicated good data for model fitting (*χ*^2^_8_=11.54, *P*=.17). Weighted scores of the 9 item factors were derived based on the β coefficients [25] obtained from the logistic regression model. The inclusion of factors CD-Acc1 (*P*=.07), CD-Acc2 (*P*=.06), TM-Acc4 (*P*=.07), RS (*P*=.06), and DR-RT2 (*P*=.05), despite their marginal significance in the regression model, was to strengthen content representativeness of EOmciSS (Table 4).

**Table 2:** Comparison of demographic characteristics of participants in the mild cognitive impairment and normal cognition function subsets in the training set (579/827, 70% of the data set).

Characteristics		Normal cognition function subset (n=402)	Mild cognitive impairment subset (n=177)	*χ*^2^ (*df*)/Z	*P* value	
Age (year)^a^		64.5 (60.00-70.00)	66.00 (62.00-71.00)	1.974 (1)	.048	
**Sex,** **n (%)**				0.170 (1)	.68	
	Male	148 (36.8)	62 (35.0)			
	Female	254 (63.2)	115 (65.0)			
Education (year)^a^		12.00 (9.00-14.00)	9.00 (9.00-12.00)	–6.818 (1)	<.001	
BMI (kg/m^2^)^a^		23.30 (21.48-25.59)	23.74 (21.70-25.29)	0.137 (1)	.89	
**Concomitant chronic disease,** **n (%)**				4.130 (2)	.12	
	Nil	205 (51.0)	78 (44.1)			
	n=1	140 (34.8)	63 (35.6)			
	n≥2	57 (14.2)	36 (20.3)			
**Smoking,** **n (%)**				0.176 (1)	.68	
	Yes	30 (7.5)	15 (8. 5)			
	No	372 (92.5)	162 (91.5)			
**Drinking,** **n (%)**				0.743 (1)	.39	
	Yes	78 (19.4)	29 (16.4)			
	No	324 (80.6)	148 (83.6)			
**Proficiency using electronic devices, n (%)^b^**				23.156 (1)	<.001	
	Yes	334 (83.1)	115 (65.0)			
	No	68 (16.9)	62 (35.0)			

^a^A 2-sample rank test was used because the data did not conform to a normal distribution. Median values (25th percentile-75th percentile) instead of mean values were used to describe results.

^b^The cutoff for yes/no was the average time of using an electronic device (eg, smartphone) for 1 hour or more/day.

**Table 3:** Logistic regression on EOmciSS^a^ item factors predicting membership of the NCF^b^ group or the MCI^c^ group in the training set (579/827, 70% of the data set).

Item factors	Regression coefficient (β)	Odds ratio (95% CI)	*P* value
CC^d^-Acc^e^1	1.590	4.904 (2.776-8.644)	<.001^f^
CD^g^-Acc1	1.663	5.273 (0.898-30.96)	.066^h^
CD-Acc2	1.177	3.244 (0.956-11.007)	.06^h^
CD-Acc3	1.331	3.786 (1.827-7.845)	<.001^f^
TM^i^-Acc1	.488	1.629 (0.486-5.461)	.43
TM-Acc2	.655	1.926 (0.583-6.362)	.28
TM-Acc3	–.197	0.821 (0.136-4.944)	.83
TM-Acc4	1.878	6.541 (0.856-49.988)	.07^h^
TM-Acc5	20.442	—^j^	.10
TM-Acc6	1.776	5.903 (0.0477-43.469)	.47
TM-OrderAcc	–21.735	—	.10
DR^k^-Acc1	1.424	4.155 (1.201-14.376)	.03^f^
DR-Acc2	.522	1.685 (0.242-11.731)	.60
DR-Acc3	.748	2.114 (0.345-12.947)	.42
RS^l^	.569	1.766 (0.973-3.207)	.06^h^
CC-RT^m^	<.001	1 (0.996-1.003)	.92
CD-RT	.001	1.001 (0.994-1.008)	.84
TM-RT	.002	1.002 (0.995-1.01)	.56
DR-RT1	.013	1.014 (1.002-1.025)	.02^f^
DR-RT2	.026	1.026 (1.000-1.053)	.05^h^
Proficiency using device^n^	.293	1.34 (0.688-2.609)	.39
Age	.036	1.036 (0.987-1.089)	.15
Years of education	–.070	0.933 (0.842-1.033)	.18
GDS-15^o^	.245	1.278 (1.117-1.461)	<.001^f^

^a^EOmciSS: Efficient Online MCI Screening System.

^b^NCF: normal cognitive function.

^c^MCI: mild cognitive impairment.

^d^CC: cube copying.

^e^Acc: accuracy.

^f^*P*<.05.

^g^CD: clock drawing.

^h^*P*=.05-.07.

^i^TM: trail making.

^j^Not applicable.

^k^DR: delayed recall.

^l^RS: reaction speed.

^m^RT: reaction time.

^n^“Proficiency using device” is a measure of proficiency of using an electronic device.

^o^GDS-15: 15-item Geriatric Depression Scale.

**Table 4:** Weighted scores of significant EOmciSS^a^ item factors based on the β coefficient values of the regression model for classification of participants with NCF^b^ and participants with MCI^c^ in the training set (579/827, 70% of the data set).^d^

Items	Regression coefficient (β)	*P* value	Weighted scores^e^
CC^f^-Acc^g^1	1.590	<.001	1.59
CD^h^-Acc1	1.663	.07	1.66
CD-Acc2	1.177	.06	1.18
CD-Acc3	1.331	<.001	1.33
TM^i^-Acc4	1.878	.07	1.88
DR^j^-Acc1	1.424	.03	1.42
RS^k^	.569	.06	0.57
DR-RT^l^1	.013	.02	0.01
DR-RT2	.026	.05	0.03

^a^EOmciSS: Efficient Online MCI Screening System.

^b^NCF: normal cognitive function.

^c^MCI: mild cognitive impairment.

^d^Significance threshold was set at *P*≤.07 to maximize the content of the 9 item factors for EOmciSS.

^e^Total weighted score is 9.67.

^f^CC: cube copying.

^g^Acc: accuracy.

^h^CD: clock drawing.

^i^TM: trail making.

^j^DR: delayed recall.

^k^RS: reaction speed.

^l^RT: reaction time.

### Verification of Discriminative Validity (Validation Set)

**Figure 5:**
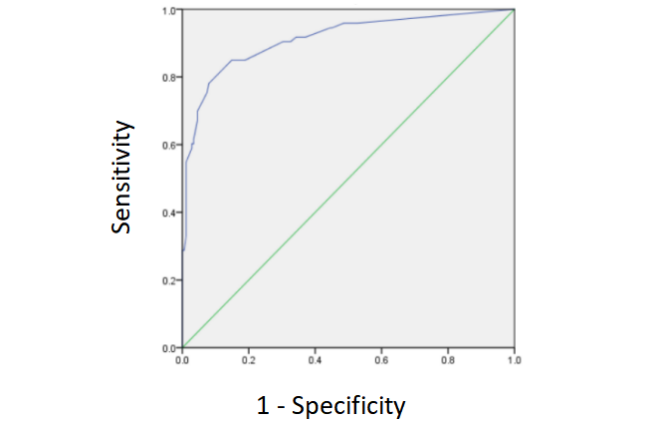
ROC curves. ROC curves for the 9 EOmciSS item factors differentiating participants with MCI from those with NCF in the validation data set. AUC for the validation set was 0.912 (95% CI 0.868-0.955; *P*<.001). AUC: area under the curve; EOmciSS: Efficient Online MCI Screening System; MCI: mild cognitive impairment; NCF: normal cognitive function; ROC: receiver operating characteristic.

The validation set comprised 248 participants. Significant between-subgroup differences were in age (*P*=.04) and number of years of education (*P*<.001). Use of an electronic device showed marginal significance in subgroup differences (*P*=.07). The MCI subgroup (2/73, 2.7%) had significantly fewer participants reporting a history of tobacco smoking than the NCF group (20/175, 11.4%; *P*=.03). Participants in the NCF subgroup showed a significantly higher mean score on the MoCA (*P*<.001) and a lower mean score on the GDS-15 (*P*=.01) than those in the MCI subgroup. Significant between-subgroup differences were not found for the mean score on the IADL scale (*P*=.34). With the same parameters derived from the training set, the AUC for the classification of the NCF and MCI subsets was 0.912 (*P*<.001; 95% CI 0.868-0.955). The cutoff score of 7.90 yielded 84.9% sensitivity, 85.1% specificity, and Youden index of 0.70 for the validation set. The receiver operating characteristic curves of the validation set are presented in Figure 5.

## Discussion

### Expected Findings

We aimed to reveal the psychometric properties of EOmciSS, a newly designed, rapid, digital cognitive test for screening of individuals with MCI in community settings. The ability of EOmciSS to differentiate individuals with MCI risks from those with NCF was found to be higher than that of other online systems in this research field. Essential content domains for the MCI classification versus the NCF classification were mainly in the Acc of the performances in the order of visuospatial attention, planning, and executive function (in CC and CD); cognitive flexibility and working memory (in TM); and verbal memory (in DR). In the TM subtest, the connection from the third-to-fourth alternative symbols contributed the most to the classification model. Besides, the speed of performances in terms of RT (in RS) and recall of memory items (in DR) significantly predicted membership of the MCI group or the NCF group. Incorporation of a “depressive mood” measure in the first section of EOmciSS is a new design for a cognitive screening test. Evidence suggests that individuals with a depressive mood are likely to have low motivation and delays in executing actions, which would pose threats to the internal validity of cognitive test performances (eg, lower Acc or longer RT). For EOmciSS, the impacts would be on the measures of Acc and RT. The identification of users of EOmciSS with the GDS-15 before them entering into the cognitive test section would safeguard the validity of the cognitive screening for MCI.

The test construct of EOmciSS was substantiated with results from the confirmatory factor analysis. The 20 item factors were clustered satisfactorily into 3 latent Acc factors with respect to the test content of visual attention and mental flexibility (TM), visuospatial and executive function (CD and CC), memory (DR), and cognitive processing speed (RS). The Acc of the 3 CD items and 1 CC item was grouped under 1 single latent factor. For CD, the 3 Acc item factors were “placing the 12 numbers (ie, “1” to “12”) on the face of a clock” (#CD-Acc1), “orienting the hour hand” (#CD-Acc2), and “orienting the minute hand” (#CD-Acc3). The CD item had 1 Acc item factor: “drawing the cube.” Within CD items, the “orienting the minute hand” Acc item factor was the most difficult (mean 0.85), but it was less difficult than the “draw the cube” item factor (mean 0.63). CD and CC are tests for visuospatial, planning, and executive functions, which have been reported to be effective for screening for individuals with MCI [50,51]. Therefore, findings by EOmciSS are consistent with those reported previously.

We emphasized the Acc and speed of participants’ performances when designing EOmciSS. EOmciSS has made possible the capture of both performance parameters at a response level rather than at an item level. For instance, CD has 3 Acc factors and 1 RT factor. The definitions for the 4 item factors are different from those in the Digital Clock Drawing study, which quantifies the size, angle, and spacing between the clock elements, and their latencies [52]. In this study, “drawing numbers” involved dragging 12 numbered icons to the designated positions on a clock face; and the “drawing hands to 10 minutes after 11 o’clock” involved dragging the hands from 12 o’clock to the specified time. Only the Acc of the 3 parts of the drawing (and not the RT) was the significant predictor of membership of the MCI group versus the NCF group. Correctly “orienting the minute hand” was found to be the most significant predictor of classification of MCI versus NCF. Our finding is consistent with the data from 2 studies (1 conventional and 1 digital) which reported that correct placement of the “minute arm” was the best predictor of amnesia in individuals with MCI [29,53]. Such comparable results support the adoption of the 3 digital CD Acc item factors in EOmciSS.

For the CC item, Oonuma et al [54] reported that 31.1% of individuals with normal cognition failed to complete the test correctly. The difficult level revealed for the single CC item in this study suggested that 37% (306/827) of the participants failed to complete the task. The ratio of failure to complete CC of participants with MCI to participants with NCF was around 5:1 (odds ratio 4.9). Such comparable results support the adoption of the design and format of the 1 CC Acc item factor in EOmciSS.

The TM items, despite their marginal significance in the prediction model, appeared to have a compatibly important role as those of CD and CC. Interestingly, among the 6 factors, the “third-to-fourth symbol connection” Acc item factor (#TM-Acc4) was the best discriminative index. The “third-to-fourth symbol connection” was the only TM item to be included in the MCI versus NCF prediction model. It yielded the largest β coefficient and, therefore, was assigned with the heaviest weight contributing to the EOmciSS score. The design of the TM item is comparable with the conventional Trail Making Test [28,55]. A previous study suggested that the Trail Making Test measures task switching and working memory [56]. The correct connection of the fourth symbol from the start of the task would demand both functions. As expected, the Acc and speed of the DR items were significant predictors of membership of the MCI group versus the NCF group. The word “fruit” yielded the highest discriminative index (0.79) among the 3 words. We speculated that this superior discriminative power may be due to learning a new task rule (ie, encoding words and keeping them in mind) and learning the first word in a 3-word series. The learning involved would have placed substantial demand on the memory system of individuals with MCI. Our speculation is supported by the findings of studies on patients with MCI who showed deficits in DR of a series of items [57,58].

About 90% (745/827) of participants completed EOmciSS in 10 minutes. This short time is a promising factor for maximizing the number of community-dwelling older adults who complete the test. Compared with other computerized tests, the CoCoSc system used by Wong et al [22] had 5 subtests, but the time taken to complete the test was 15 minutes. The CoCoSc system has a lower ability (78% sensitivity and 69% specificity) for screening MCI and NCF. Groppell et al [59] developed the BrainCheck Memory test. It has 6 subtests and takes about 21 minutes to complete. The sensitivity of the BrainCheck Memory test is 89% and specificity is 78%. By comparison, EOmciSS required a shorter time for completion but had relatively promising sensitivity (84.9%) and specificity (85.1%) for screening of MCI and NCF.

Our study had 3 main limitations. First, participants were older adults who had been educated for more than 6 years. Thus, our results may not be generalizable to those who have only a few years of education or who are illiterate. We should explore possible adaptations of test content and a user interface that is appropriate for these groups. Second, compared with other computerized screening tests, EOmciSS could be used to differentiate individuals with MCI from individuals with NCF. However, the content representativeness of EOmciSS was limited. Therefore, EOmciSS data should be interpreted as indicating the risks, but not the diagnosis, of MCI. Additional neuropsychological and medical tests are recommended for reaching a clinical diagnosis. Third, we used a cross-sectional method to gather evidence, which hampers the repeatability and stability of test results. We must investigate the robustness of the test scores and classification results by using a longitudinal design and analytic methods, as described by Lei and colleagues [60].

### Conclusions

EOmciSS is a new, computerized, cognitive screening test designed for use by community-dwelling older adults. The test content, user interface, and administration method enable older adults to complete the test with a smartphone or tablet within 10 minutes. The test constructs of EOmciSS can be factored into Acc and RT components, both of which contributed significantly to the ability of EOmciSS to differentiate individuals with MCI risks from those with NCF. The screening ability of EOmciSS was revealed to be the strongest among other computerized tests, with comparable test constructs and time needed for completion. EOmciSS could serve as a first-line test for identifying individuals with MCI risks in the community. Application of EOmciSS could enable rapid, inexpensive, large-scale screening among older adults. Individuals who receive positive screening results should be followed up with more comprehensive neuropsychological and medical tests before confirmation of an MCI diagnosis.

## Appendix

Multimedia Appendix 1 Descriptions of the Participants.

Multimedia Appendix 2 Descriptions of test content of EOmciSS (Efficient Online MCI Screening System).

## Abbreviations

Acc: accuracy
AD8: Ascertain Dementia-8
AUC: area under the curve
CANS-MCI: Computer-Administered Neuropsychological Screen for Mild Cognitive Impairment
CC: cube copying
CD: clock drawing
C-MoCA: Chinese version of the Montreal Cognitive Assessment
CoCoSc: Computerized Cognitive Screen
DR: delayed recall
EOmciSS: Efficient Online MCI Screening System
GDS-15: 15-item Geriatric Depression Scale
IADL: Instrumental Activities of Daily Living
MCI: mild cognitive impairment
MoCA: Montreal Cognitive Assessment
NCF: normal cognitive function
ROC: receiver operating characteristic
RS: reaction speed
RT: reaction time
TM: trail making
