# Supplementary material for: An Effective Test (EOmciSS) for Screening Older Adults With Mild Cognitive Impairment in a Community Setting: Development and Validation Study
Source: J Med Internet Res. 2023 Jan 30;25:e40858. doi: 10.2196/40858 (PMC9926348; doi:10.2196/40858)
Supplement: Multimedia Appendix 1 [file jmir_v25i1e40858_app1.docx]

**Multimedia Appendix 1**

**Descriptions of the Participants**

A total of 1,584 older adults were recruited to participate in the study. They were all community-dwelling individuals living in the Fuzhou city. One-hundred and fifty-one older adults did not meet the inclusion criteria because they were not within the 55 to 75 years old range set for this study. Another 202 older adults were excluded as 13 of them presented with drug or alcohol abuse problems, 29 reported history of suffering from cerebral infarction or traumatic brain injury, 116 scored 8 or above on the EOmciSS Section 1 - GDS-15 screening for potential depressive mood, seven scored within the ranges of potential dementia according to the MoCA or AD-8, and 37 for other reasons. The other 150 older adults did not manage to complete the EOmicSS.

Among the 1,081 who met the inclusive and exclusive criteria, and completed the EOmciSS. One-hundred and ninety-six participants were later excluded from the final dataset as they were verified to receive less than six years of education. Fifty-eight participants showed excessive missing data from the test results. The final sample size entered the analyses was 827 participants.


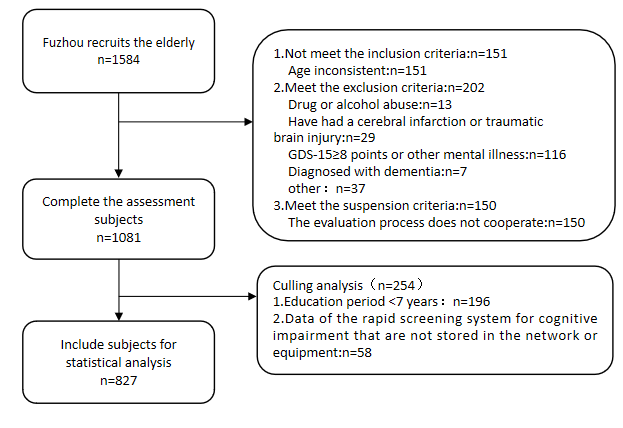


Figure: Flow diagram of the study
