# Supplementary material for: An Effective Test (EOmciSS) for Screening Older Adults With Mild Cognitive Impairment in a Community Setting: Development and Validation Study
Source: J Med Internet Res. 2023 Jan 30;25:e40858. doi: 10.2196/40858 (PMC9926348; doi:10.2196/40858)
Supplement: Multimedia Appendix 2 [file jmir_v25i1e40858_app2.docx]

**Multimedia Appendix 2**

**Descriptions of Test Content of EOmciSS**

**General:**

The Efficient Online Mild Cognitive Impairment Screening System (EOmciSS) is designed to identify individuals who have significant risks of mild cognitive impairment (MCI) among community-dwelling older adults. The screening test is operated via tablets or mobile phones and normally take less than 10 minutes to complete. The EOmciSS has two sections. The first section is for screening of depressive mood, while the second section is for screening of cognitive functions. For the first section on depressive mood, the EOmciSS adopts the 15-item Geriatric Depression Scale. For the second section on cognitive functions, there are five subtests tapping on impairments common to manifest among MCI individuals. The five subtests have a total of test 20 items. The scoring of the 20 items is based on accuracy (13 items), speed (six items) or order (one items) performance. Participant will need to pass the first section depressive mood screen (GDS, cut-off < 8 score) before entering in the second section cognitive screen. The results of the depressive mood and/or cognitive screens are summarized by the end of the EOmciSS operation. Below are the details of each of the sections and subtests of EOmciSS.

**Section 1 - Depressive mood**

The 15-item Geriatric Depression Scale (GDS) is to identify participants who are presented with significant depressive mood symptoms. Positive response to one statement item has one score. The maximum score is 15, and the cut-off score for not showing potential risk of depressive mood is “< 8”. The participants will be informed of the result of the depressive mood screen and be recommended to seek medical advice for the potential risks of depressive mood if the score is 8 or above. The EOmciSS will then be terminated.

**Section 2 - Trail Making (TM)**

The Trail Making test has six items, which are designed to assess visual attention and mental flexibility functions. The participant is to use a hand to connect Arabic “1” to Chinese “一”, Chinese “一” to Arabic “2”, and Arabic “2” to Chinese “二” till the alternative forms of numbers end at Chinese “四”. The difficulty levels of the TM test increases with the greater the numbers as the increasing number of alternative moves between the two forms would demand higher level of attention and sustaining mental flexibility, i.e., from 1^st^ to 6^th^ items. The participant’s performances are measured in terms of the accuracy and speed of each connection made. The accuracy factors are correctly to connect 一-to-2, 2-to-二, 二-to-3, 3-to-三, 三-to-4, 4-to-四. The speed factor is defined as the total time taken to make all the correct connections in the six test items. Besides, there is an order factor, which is defined as the correct order made across all the items. The accuracy factor scores 6 points, one score for one accurate performance. The speed factor scores 1 point, scored when the total completion time is shorter than the average plus one standard deviation. The sequence factor scores 1 point, scored when the entity order was completed correctly. Thus, this test has a total of 8 performance factors for a total of 8 points.In sum, there are eight performance factors in this subtest including six for accuracy, one for speed, and one for order.

**Section 2 – Clock Drawing**

The Clock Drawing test has three items, which measures visuospatial and executive functions. The participant is to use a hand to drag 12 number icons of “1” to “12” at the right side of the screen to the appropriate positions on the face of a clock appear at the left side of the screen. The placing of the 12 numbered icons completes the first item. Afterward, the participant is to move the hour-hand and minute-hand on the face of the clock from their original positions to positions representing “ten minutes past ten o’clock” (i.e., 10:10). The positioning of the hour and minute arms completes the second and third item of the test. Difficult level of the dragging the numbered icons on the clock face increases with the numbers of the icons correctly placed. The positioning of the minute-hand would be more challenging than that of the hour-hand because it involves the five-minute concept for the former (i.e., icon “2” representing 10 minutes). Accuracy is a performance factor for all the three items. It is defined as the correct positioning of all the 12 icons on the clock face (1 score), and the correct positioning of the hour-hand (1 score) and minute-hand (1 score). Speed is also a performance factor defined as the total time taken to complete all the three items (1 score when The total time is shorter than the average total time plus one standard deviation). In sum, there are four performance factors in this subtest including three for accuracy and one for speed.

**Section 2 – Cube Copying**

The cube copying test has one item which measures visuospatial and executive functions. The participant is to refer to a three-dimension cube figure shown on the screen and reproduce the same in the space next to the figure. The copying does not require the participant to draw with free hand. Instead, the copying requires the participant to use a hand to connect the 13 white dots on a blue background located in the same positions with the corners of the cube figure. Accuracy is a performance factor defined as correct connecting all the dots to form a cube (1 score). Speed is also a performance factor defined as the total time taken to complete the copying (1 score when the total time is shorter than the average total time plus one standard deviation). In sum, there are a total of two performance factors in this subtest including one for accuracy and one for speed.

**Section 2 – Reaction Time**

The reaction time test has one item which measures cognitive processing speed. The participant is to tap on the triangular objects presented randomly on the screen as soon as possible with a finger. A triangular object will disappear once after the tapping by the participant. The duration of the triangle tapping is 30 seconds. Speed is a performance factor defined as the average time spent tapping on each triangle by the participant(1 score when the average time is less than 0.2 seconds). In sum, there is one speed performance factor for this subtest.

**Section 2 – Delayed Recall**

The delayed recall test has three item which measures memory function. The participant is to view three objects presented on the screen in sequence at the beginning of the Section 2 EOmciSS. The participant is reminded to recall them later. By the end of Section 2, the participant is to identify from nine objects those they viewed before by clicking on the icons appeared on the screen. Accuracy is a performance factor defined as the number of object(s) correctly selected from the list (1-3 Score). Speed was also a performance factor, defined as the total time to complete the memory at the beginning (1 score when the total time is less than the average total time plus one standard deviation) and the total time to select a response at the end (1 score when the total time is less than the average total time plus one standard deviation). In summary, there are five performance factors in this subtest, including three accuracy and two speed factors.
